# Perspectives on long pentraxin 3 and rheumatoid arthritis: several potential breakthrough points relying on study foundation of the past

**DOI:** 10.7150/ijms.54787

**Published:** 2021-03-03

**Authors:** Cheng Qiu, Yichao Han, Hanwen Zhang, Tianyi Liu, Haodong Hou, Dan Luo, Mingzhi Yu, Kai Bian, Yunpeng Zhao, Xing Xiao

**Affiliations:** 1 Department of Orthopedic Surgery, The First Affiliated Hospital of Shandong First Medical University, Jinan 250014, Shandong, P. R. China.; 2 Cheeloo College of Medicine, Shandong University, Jinan 250012, Shandong, P. R. China.; 3 Department of Orthopaedic Surgery, Qilu Hospital, Cheeloo College of Medicine, Shandong University, Jinan 250012, Shandong, P. R. China.; 4 College of Stomatology, Qingdao University, Qingdao 266071, Shandong, P. R. China.; 5 Key Laboratory of High Efficiency and Clean Manufacturing, School of Mechanical Engineering, Shandong University, Jinan 250061, Shandong, P. R. China.

**Keywords:** Pentraxin 3, Rheumatoid arthritis, Synovitis, TNF-α, Inflammation

## Abstract

Rheumatoid arthritis (RA) is a systemic chronic autoimmune inflammatory disease which is mainly characterized by synovitis and results in a severe burden for both the individual and society. To date, the underlying mechanisms of RA are still poorly understood. Pentraxin 3 (PTX3) is a typical long pentraxin protein which has been highly conserved during evolution. Meanwhile, functions as well as properties of PTX3 have been extensively studied. Several studies identified that PTX3 plays a predominate role in infection, inflammation, immunity and tumor. Interestingly, PTX3 has also been verified to be closely associated with development of RA. We therefore accomplished an elaboration of the relationships between PTX3 and RA. Herein, we mainly focus on the associated cell types and cognate cytokines involved in RA, in combination with PTX3. This review infers the insight into the interaction of PTX3 in RA and aims to provide novel clues for potential therapeutic target of RA in clinic.

## Introduction

Rheumatoid arthritis (RA) is a common autoimmune disease that afflicts 0.5-1% population all over the world 1. Hitherto, the mechanism of RA is still poorly understood, while environmental factors and genetic causes may both contribute to the morbidity of RA 2. Autoantigen should be considered as the crucial clue due to the nosogenesis in autoimmune diseases, including RA. Collagen type II, the chief contained protein of hyaline cartilage, has been recognized as a critical autoantigen 3. Meanwhile, collagen induced and collagen-antibody induced autoimmune inflammatory arthritis mouse models have become an instrument to identify potential treatment against RA 4, 5. RA is characterized as a chronic inflammation procedure, and the involved joints are infiltrated by inflammatory cells 6. Therefore, the construction of inflammatory microenvironment in joints benefits from inducible lining of cell types sites. Provocative inflammation leads to gradual cartilage destruction and progressive bone erosion 7.

The classical long pentraxin, pentraxin 3 (PTX3), is expressed and released by various cell types in response to inflammatory cytokine stimulation and microbial invasion 8-10. PTX3 is affiliated with the soluble pattern recognition receptors with high conservative structure both in mice and human during evolution 11. Currently, persistent studies have acknowledged that PTX3 plays an indispensable role in immunity, inflammation, infection, tissue remodeling and fertility 12.

Herein, we mainly sum up the roles of PTX3 in RA mingling with our own insights. By virtue of accumulative evidence, we recognize that PTX3 may play a crucial role in RA progression. On this stage, we expect that future studies may focus on these clues provided by us to hopefully elucidate more about the mechanisms of RA.

## The structure of PTX3

**Figure 1:**
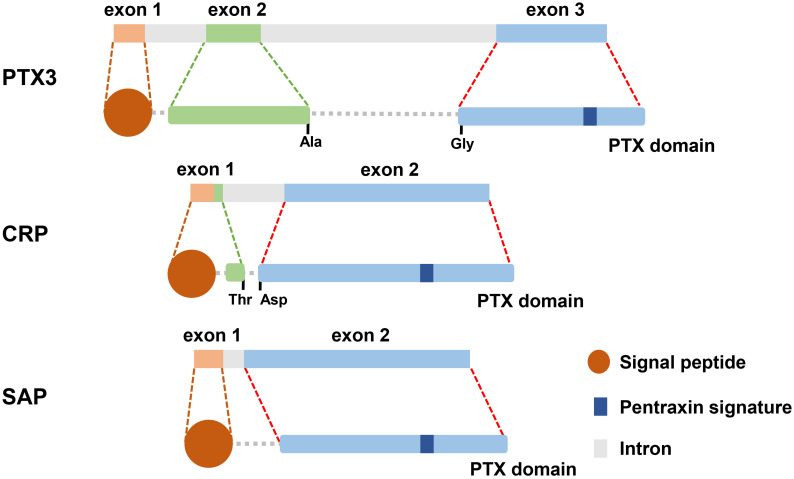
** The gene and protein structure of PTX3, CRP and SAP.** What is known is that all three molecules are secreted protein. There are 3 exons located on PTX3 gene which is different from 2 exons on the genes of CRP and SAP. Preliminary peptides of PTX3, CRP and SAP are endowed with signal peptide located on their N-terminal domain. Long PTX3, along with CRP and SAP, is characterized by pentraxin signature which is the pentraxin family sign.

Pentraxins are recognized as a category of ancient protein superfamily with a highly conservative structure in evolution 13. The pentraxins are manifested as their carboxy-terminal with an identical 203 amino acid pentraxin domain 14. According to the primary structure of the subunit, they can be classified as two groups: long pentraxins and short pentraxins 14. The classical short pentraxins, C-reactive protein (CRP) and serum amyloid P (SAP) component, are acute-phase proteins major produced by liver in response to inflammation, whereas PTX3 is the typical prototype of long pentraxin proteins **(Figure 1)** 15-17.

PTX3 contains 381 amino acids, including a 17-amino acid signal peptide, which is characterized by a sheet-like secondary structure and a spiral arrangement of domain 18. However, SAP consists of calcium-dependent ligand binding and a unique flat β-film structure similar to leguminous lectins 19. And CRP is composed of five non-covalently bound precursors arranged symmetrically around the central hole. The CRP promoter has 206 amino acids and is folded into two anti-parallel slices with a flat jelly topology 20.

The primary sequence of PTX3 is highly conserved during evolution. PTX3 protomer is assembled into an N-terminal region and a 203 amino acids long C-terminal domain, and then formed multimers are stabilized by disulfide bonds 21. Two cysteines are responsible for determining the secondary structure of the domain at amino acid positions 210 and 271 to form the disulfide bridge 22. The C-terminal domain (203 amino acids) of PTX3 has homology with the classic short pentraxins (8 amino acids in length) and is conserved among all members of the pentraxin family 15, 21. Currently, the functions of the N-terminal domain (178 amino acids) of PTX3 are still unidentified 23.

## PTX3 in innate immunity

PTX3, a soluble pattern recognition molecule (PRM), is a key factor in humoral innate immune response to microorganisms 24. Pentraxins and complement system can opsonize apoptotic cells, promptly facilitating the uptake of cellular debris to control the maintenance of peripheral immune tolerance 25. Meanwhile, PTX3 interacts with a variety of ligands including growth factors, extracellular matrix components and selected pathogens 26. The complement C1q is the first characteristic ligand described by PTX3 14. The plastic-immobilized C1q binds to PTX3, which also interacts with the C1q spherical head, especially the charged residues located at the top of the molecule 13, 27. PTX3 directly regulates the activation of the classical complement cascade by combining with C1q, in addition to activating indirect complement mediated immune response 14. Intriguingly, PTX3 also blocks the relevant site of action by binding to C1q in the liquid phase to inhibit complement activation 13. Moreover, PTX3 also interacts with the factor H (FH) and C4 binding protein (C4BP) to adjust the complement cascade 24. Clearance of apoptotic cells is related to the interaction of PTX3 and C1q 16. The glycosylation of PTX3 may abrogate the interaction between PTX3 and C1q, and subsequent PTX3-mediated complement activation is then blocked 21.

Toll-like receptor (TLR) participates in the production of C1q and PTX3 by immature dendritic cells (imDCs) 28. During acute phase immune response, imDCs, which produce phagocytosed antigens, execute antigen presentation regulated by PTX3. Antimicrobial resistance-related functions of PTX3 are related to the interaction of conserved ancestral domains in innate immunity 17, as well as tissue repair 24. PTX3 has been proven as the opsonin for bacteria and fungi, promoting medullary cell recognition and phagocytosis in a complement- and FcγR-dependent manner, as well as neutralizing viral infections 15. For instance, the neutrophil derived protein PTX3 is essential for controlling fungal growth 14.

To date, there are numerous findings on the role of PTX3 in innate immune system. In conclusion, PTX3 facilitates the recognition of microbial moieties and the activation of complements. Additionally, PTX3 plays a role in the activation of some adaptive immune responses, including macrophage polarization and DC maturation.

## Role of PTX3 in related inflammation

The extraordinary role of PTX3 in inflammation has been identified by various studies. Herein, we mainly summarize the classic types of inflammatory responses in which PTX3 involved, comprising cancerous inflammation, retinal inflammation, metabolic inflammation, vascular inflammation, urinary tract inflammation and airway inflammation below **(Table 1)**.

## PTX3 in cancerous inflammation

Based on uniform concept of various insights, the occurrence of tumor is highly associated with the co-function of multi-factors. The infiltrated acclimation of immune cells in intricate tumor microenvironment is a typical characteristic for neoplasm progression. Chronic tumorous inflammation largely encourages cancer cells to survive and proliferate, and adjacent molecules including selectins and chemokines with their receptors, are co-opted by cancer cells for migration or metastasis 29.

Pentraxins are associated with the regulation of chronic tumorous inflammation and play a governing role in tumor development 29, 30. Among the entire pentraxins family members, PTX3 is implied to achieve reduced expression in several malignant cancers' progressions. Human esophageal squamous cell carcinoma, selected mesenchymal and epithelial tumors were reported to be related with the hypermethylation of PTX3 promoter, with subsequent diminished production 31. Meanwhile, cancer cells succumbing to inhibition were observed using a methylation inhibitor, 5-Aza-2'-deoxycytidine, which could sustain PTX3 expression, implying that PTX3 may serve as a potential therapeutic target 32. Furthermore, 12-Dimethylbenzo pyrene/retinoic acid treatment is associated with mesenchymal tumor and dermal cancer, and the susceptibility of *Ptx3*^-/-^ mice to those is greatly increased. In-depth analysis of two carcinogenic models suggested that PTX3 deficiency was involved in more pronounced inflammation, infiltrating macrophages, pro-inflammatory cytokines, pronounced neovascularization and increased C3 deposition and C5a levels. Furthermore, tumors that occur to *Ptx3*^-/-^ mice are mostly distinguished by *trp53* mutations and oxidative DNA damage 30. The shortage of PTX3 also decreases FH localization, but increases C3 deposition and tissue damage. Lack of C3 or inhibition of CCL2, followed by a decrease in tumor infiltrating macrophages, is sufficient to restore the phenotype observed in *Ptx3*^-/-^ mice 30, 33. Therefore, PTX3 may be assumed to play a protective role for carcinogenesis in inflammatory tumor microenvironment relying on regulation of complement activation.

Recently, a novel therapeutic strategy for cancers is raised by virtue of overexpression of PTX3 which inhibits fibroblast growth factor-2 (FGF2) dependent tumor growth 34. PTX3 induced inflammatory compartment production may be similarly involved in these processes.

## PTX3 in retinal inflammation

Retinal inflammation plays a key role in the development of age-related macular degeneration (AMD), which deposits the complement system in the acute phase of the macula, leading to inflammatory disorders. Complement protein FH binds to CRP, as well as PTX3 that down-regulates inflammatory response. The truncated form of the unknown FH, which is also called factor F-like protein 1 (FHL-1), may have independent immunoregulatory functions in the human retinal environment 35. PTX3 is present in the macula of human eyes, and the polymorphism of complement factor H Y402 (Y402H) related to AMD alters the binding of FHL-1 to PTX3 36. In acute stage, pentameric protein CRP deposites in the macula, which activates the complement system and leads to dysregulation of inflammation. Taking into consideration of the interplay between PTX3 and FH which partly is caused by the binding site of CCP7, similarly integrated with CRP. As expected, the solid phase binding experiments were verified by showing the interaction of full length FH with immobilized PTX3 in relevant studies, and it was also proved that FHL-1 also interacts with PTX3 under the same conditions. In human donor ocular tissues, PTX3 binds to FH on CCP7, CCP19 and CCP20, which is capable of regulating the classical pathway of complement and the lectin pathway, and local expression at the site of inflammation 37. It is worthy of note that PTX3 enhances the interaction of FH with apoptotic cells, resulting in a regulatory enhancement of iB3b 36. However, plasma levels of PTX3 and its gene expression in retinal pigment epithelial cell (RPEC) and choroid are both independent of AMD status. Interestingly, PTX3 expression was increased in cultured RPEC which was stimulated with inflammatory cytokines or lipid peroxides. Furthermore, the genetic defect of PTX3 was amplified in an animal model of AMD that increased both C3a and IL-1β levels in RPEC and macrophage accumulation in the choroid *via* complement activation 38. Therefore, it could be inferred that PTX3 regulates the retinal inflammatory response possibly by binding to FHL-1.

## PTX3 in metabolic inflammation

Obesity is usually the result of an imbalance of excess nutrients and metabolic homeostasis, leading to a chronic inflammatory state and an increase in systemic proinflammatory mediators and adipose tissue-retaining immune cells phenotype from tolerant to pro-inflamed. Once the immune cells in adipose tissue transform into proinflammatory phenotype, they will produce adipokines and inflammatory cytokines 39. These factors promote the recruitment of immune cells, allowing the persistence of local and systemic inflammation. Progressive accumulation of fat may result from excessive nutrition, elevated free fatty acids, inflammation and activation in adipose tissue. This further restrains inflammation of adipose tissue and insulin resistance 39. The absence of PTX3 shows the phenotypic polarization of M2 macrophages, which retains the macrophages of adipose tissue, thereby preventing the accumulation of lipid and inflammation in obesity caused by high-fat diet 40. Related experiments also demonstrated that PTX3 controls angiogenesis by binding to FGF2. These results also showed a direct correlation between expression of vascular endothelial growth factor (VEGF), adipose tissue angiogenesis and M2 macrophage activation 30. Similarly, PTX3 deficiency is associated with increased expression of CD31 and VEGF in adipose tissue, suggesting an improvement in vascular network, which may also contribute to reduced accumulation of lipid in *Ptx3*^-/-^ mice 30, 41.

## PTX3 in vascular inflammation

Vascular inflammation plays an important role in vascular homeostasis and restricted immunity of organs and tissues in the whole body, reflecting the dynamic interaction of circulating cells, blood molecules and vascular structure 42. PTX3 is mainly produced in the inflammatory sites as a crucial orchestrator for regulation of inflammation. Neutrophils release PTX3 at an early stage, while the synthesis of endothelial cells and macrophages is to maintain the production of PTX3 43. PTX3 inhibits the phagocytosis of neutrophils in late apoptosis and promotes their accumulation in the vascular wall in vascular stenosis, such as small vasculitis. The inadequacy of PTX3 is associated with increased accumulation of macrophages and exaggerated monocytes in atherosclerotic lesions. In addition, *Ptx3^-/-^* mice showed adhesion molecules on the vessel wall, and increased expression of cytokines and chemokines, thus indicating that PTX3 regulates vascular-related inflammatory responses 44. Preliminary findings indicated that PTX3 with the potential pathogenic capacity in cardiovascular disease, was described as an early marker of major local activation of innate immunity and inflammation of vascular wall 45. In addition, ascending PTX3 in cardiovascular disease may show protective physiological response, which might be related to the severity of the disease 46.

## PTX3 in urinary tract inflammation

The pathogenicity of urinary tract infection (UTI) is an influential causation of urinary tract inflammation, and host immunity emerging in the urinary tract is an indispensable pathophysiological feature in urinary tract infections 47, 48. PTX3 is a humoral PRM for urinary tract infection caused by pathogenic Escherichia coli (E. coli), which is one of the major reasons for human UTI 48. PTX3 produced by urothelial cells and renal cells plays an important role in controlling urinary tract inflammation, and the deficiency of PTX3 may lead to excessive inflammation 48. Local and rapid expression of PTX3 stimulated by pathogenic E. coli is inhibition of TLR4-MyD88 pathway during UTI, aiming to enhance the recruitment of neutrophils to fight against the bacteria 49. In related studies, PTX3-deficiency is highly associated with increased both severity of infection and sensitivity to pathological inflammation. Notably, *Ptx3^-/-^* mice expressed higher levels of bacterial load in bladder and kidney, neutrophil infiltration, chemokine levels and tissue damage 50. Therefore, PTX3 may play a positive role during urinary tract inflammation.

## PTX3 in airway inflammation

Airway inflammation is characterized by infiltration of inflammatory cells and airway hyperresponsiveness, and then chronic inflammation mediates subsequent airway remodeling 51. Recent study revealed that PTX3 is highly related to allergic inflammation from asthmatic patients and ovalbumin-induced mice model. Furthermore, augmented airway response mediated by Th17-dominant CD4^+^ T cells secreting IL-6 and IL-23, which were sustained by DC cells, was observed in *Ptx3^-/-^* mice when exposed to ovalbumin (OVA) stimulation. The depletion of PTX3 promotes the phenotype of deregulated IL-2 and reprograming CD4^+^ T cells 52. Several studies indicated that PTX3 produced by epithelial cells has a regulatory effect on airway smooth muscle cell (ASMC) and is churned out in airway smooth muscle bundles and epithelial cell layers, in parallel to inflammatory cell infiltration 53. Primary human ASMC produces higher levels of PTX3 at baseline with TNF stimulation 54, while higher expression of PTX3 induces ASMC migration and promotes airway remodeling 55. Recent reports indicated that PTX3 inhibits FGF2-induced airway smooth muscle migration 56. It is possible that PTX3 may exert potential dual influence by enhancing local airway chemokine expression in human ASMC, while counteracting FGF2-induced human ASMC migration to down-regulate airway remodeling.

## Manifestations of rheumatoid arthritis

RA is an autoimmune chronic inflammatory disease that has the characteristics of joint swelling, pain, progressive cartilage damage, destruction of bone and systemic complications, even leading to severe disability 57. The main pathological aberrance of RA in joint may attribute to hyperplastic synovium, brutal bone erosion and cartilage destruction. At present, antibodies against cyclocitrulline peptide (ACPA) is a more relative biomarker, compared to rheumatoid factor (RF), that predicts invasive disease. Effective treatment can reduce the concentration of RF and ACPA 58. In RA, several studies demonstrated that inflammatory cytokines can trigger the transition from systemic immunity to arthritis and sustain a detectable proinflammatory environment, and in some cases cytokine interactions prevent inflammation from subsiding 59. Pro-inflammatory cytokines such as interleukin-1beta (IL-1β) and tumor necrosis factor-alpha (TNF-α) released from the inflamed and damaged tissue of the local inflammatory response, and subsequent secretions including histamine, serotonin, bradykinin, ATP, prostaglandin E2 (PGE2) and protons (H^+^) play a predominant role in RA. The focus of treatment is shifted to early intervention in the process of disease development to prevent inflammatory joint damage 60.

Currently, to further interpret the underlying mechanisms of RA is fundamental for both intervention and treatment of RA patients. Overall it still remains to be devoted to exploring drugs or cure treatments with significant effects for RA and patients will benefit from amelioration of this malady in terms of current situation.

## PTX3 in the pathogenesis of RA with main cell types

### Synoviocyte

Synovitis is the major manifestation in RA. PTX3 expressed by numerous cell types after stimulation with cytokines (e.g. IL-1β and TNF-α), TLR agonists or microbes in inflammation is found to be increased in rheumatoid arthritis synovial fluid. Additionally, it is known that PTX3 is also expressed in synoviocyte *in vitro* 61. PTX3 is mainly expressed in type B synoviocytes of arthritic patients with the stimulation of TNF-α, but not IL-1β 62. On the other hand, PTX3 expressed highly by synoviocytes from RA is not affected by anti-TNF antibodies, IL-1 receptor antagonists or a combination of both 61. However, synoviocytes from RA patients were implied to produce higher levels of PTX3 *in vitro* while inhibited by transforming growth factor-beta (TGF-β) and interferon-gamma (IFN-γ) 63. Yokota *et al* found that simvastatin significantly impairs RA process, and they further identified that simvastatin down-regulates the expression of monocyte chemoattractant protein-1 (MCP-1) and PTX3 in fibroblast-like synoviocyte derived from RA patients 64. Furthermore, Satomura *et al* also discovered that the acute-phase reactant, serum amyloid A (SAA), had the efficacy of induction of PTX3 in synoviocyte 65. In synoviocyte, FGF-2 is reported to play a dramatic role in enhancement of alkaline phosphatase (ALP) activity, the expression of chondrogenic genes (Sox9, Col2α1 and Aggrecan), osteogenic genes (Foxc2, osteocalcin and Col1α1) and VEGF-A, while suppressed by PTX3 addition 66. Recently, Zhao *et al* demonstrated that PTX3 suppresses the progression of RA by inhibiting the autocrine and paracrine stimulation of FGF-2 on synovial fibroblast 67.

### Chondrocyte

The original ingredient of cartilage is exclusively generated by chondrocyte. Chondrocytes involved in inflammatory microenvironment are prone to death mostly presented as apoptosis 68, bringing about the homeostasis disruption in cartilage. The detectable level of PTX3 is implied in chondrocyte. Furthermore, Haglund *et al* reported the activation of TLR4 was induced by lipopolysaccharide (LPS) that significantly promotes the production of PTX3 and other inflammatory cytokines in articular chondrocytes 69. Additionally, Barksby *et al* also indicated the genes of PTX3, IL-8 and several matrix metalloproteinases (MMPs) were up-regulated with stimulation of IL-1α along with oncostatin M in chondrocyte 70. The direct interaction with inducible expression of PTX3 in chondrocyte may mainly rely on the proinflammatory cytokine stimulation. In addition, it is well known that FGF-2 is a stimulator for chondrocyte proliferation upon cartilage wounding 71. However, whether PTX3 potentially serves as an antagonist for FGF-2 to inhibit chondrocyte proliferation is unknown.

### Macrophage

The notable function of PTX3 is possible to antagonize macrophage infiltration directly during inflammatory process. *Ptx3* knockout mice are used to improve the susceptibility of macrophage (CD68^+^) infiltration in 3-methylcholanthrene injection site 30. Also, the expression of PTX3 was evaluated to overexpress in fibrosarcoma, whereas the recruitment of infiltrated macrophage was decreased, as well as neovascularization 72. And, other evidence demonstrated that PTX3 acted as a potent chemotactic agent through interaction with neuropeptide Y to enhance the migration of macrophage indirectly 73. The powerful phagocytosis of macrophages is highly embodied in gouty arthritis, and PTX3 contributes to promoting the uptake of monosodium crystals for macrophages via increasing IL-1β and chemokine CXCL1 production 74. Previous assessment identified that distinct phenotypic macrophages are emerged in both synovium lining and cartilage tissue, and the polarization of macrophage also exists in RA joints with significant production of TNF-α and IL-1β 75. The role of PTX3 is in line with the macrophage skewing which results in increased M1 macrophage polarization and diminished M2 macrophage, whereas the opposite of PTX3 deficiency 39. At the same time, up-regulated PTX3 also functioned in promoting the macropinocytosis of M1 macrophages for low-density lipoprotein (LDL) 76. IL-10 is a common marker of M2 macrophages, assuming that the regulatory bridge may appear in the confrontation between M1 and M2 macrophages, and the role of IL-10 has been elucidated to stimulate the production of PTX3 26. The production of PTX3 and IL-10 is elevated in macrophages in response to PGE2 77.

### Dendritic cell

Dendritic cell (DC) is a typical professional APC with the function of immune tolerance and T cell activation in the pathogenesis of RA. Both C1q and PTX3 are produced by imDC in response to TLR engagement 78. Moreover, PTX3 plays a critical role in recognition of diverse pathogens and modulation of complement activation via connecting to C1q and DCs' recognition of pathogens 79. Moreover, during the inflammatory conditions of RA, exaggerated apoptosis of cells is a common feature and subsequent necrosis debris release is emerged once apoptotic cells cannot be cleared promptly 80. PTX3 hereof binds to apoptotic cells, inhibiting its recognition by DC instead of macrophage, and then embarks on normal binding late in the apoptotic process the same as binding to apoptotic cells better than necrotic ones *in vitro* 17. Furthermore, PTX3 serves as an opsonin by enhancing the phagocytosis of DCs. It inhibits the release of both TNF-α and IL-10 by LPS-challenged DCs, but the inhibitory effect is blocked in the presence of apoptotic cells 81. PTX3 suppresses the phagocytosis of DCs and the expression of immunosuppressive cytokines in order to control cross-presentation of self-epitopes expressed by apoptotic cells, promoting innate immune response to pathogens. Taken together, PTX3 presumably influences the production of such chemokines and pro-inflammatory cytokines in the synovium of RA patients by targeting on DCs.

### Osteoblast and osteoclast

Similarly, PTX3 increases the potential of osteoblast precursors to differentiate to osteoclasts by affecting the receptor activator of nuclear factor-κ B ligand (RANKL) mediated production of precursor osteoblasts 82. Furthermore, PTX3 enhances the expression of RANKL, leading to upregulated precursor osteoblasts, which causes the excessive formation of osteoclasts. In the inflammatory bone microenvironment, PTX3 influences TNF-α by inducing precursor osteoblasts to produce RANKL, which promotes the formation of osteoclasts and subsequent bone erosion 83. PTX3 may mainly manipulate the RANKL/osteoprotegerin (OPG) ratio on osteoblast precursor, instead of affecting osteoclastogenesis directly.

### Neutrophil

PTX3 is positively expressed in neutrophils progenitor cells (promyelocytes, myelocytes and metamyelocytes) during neutrophils maturation 84. Immune signals from cytokines caused neutrophils to rapidly release performed PTX3 from secondary particles. On the other hand, PTX3 prevents excessive recruitment of neutrophils to inflammatory sites 85. Incremental overexpression of PTX3 and reactive oxygen species (ROS) in neutrophils is associated with impaired endothelial function, and it may be a marker of inflammation in hemodialysis patients 86. Furthermore, neutrophils with PTX3 deficiency are prone to the detriment of fungi and they may increase the risk of invasive aspergillosis in patients when treated with hematopoietic stem cell transplantation 87. Notably, neutrophils migrate to the joint cavity early and play a role in inflammation in RA.

### Lymphocyte

It is known that PTX3 plays an indispensable role in the regulation of CD4^+^ T lymphocyte-mediated inflammation 52. The lack of PTX3 promotes a Th17-dominated CD4^+^ T cell response in the lung and enhances the survival of CD4^+^ T cell 52. Furthermore, it is suggested that PTX3 inhibits the cross-expression of antigens derived from apoptotic cells on CD8^+^ T cells 17. Given the ability to recognize limited sets of microbial molecular patterns, pentraxins are considered to be ancestors of antibodies produced by follicular B cells 11. Subsets of effector T cells, such as CD4^+^ T cells (TH_17_) that produce IL-17, play an important role in the pathology of RA, and the role of Treg in peripheral immune tolerance in RA patients remains in dispute 88. To date, a pathogenic role of these cells may contribute to the development and activity of RA and the therapy for autoimmune diseases is still largely dependent on nonspecific immunosuppression.

## Pentraxin 3 with cardinal RA-involved molecule ensemble

### TNF-α

The classical cytokine, TNF-α, is identified to exhibit a pleiotropic effect on immunity and inflammation. The majority of TNF-α originates from monocytes, macrophages, DC cells, activated B lymphocytes and T lymphocytes, which can bind to two receptors (TNFR1 and TNFR2) 89. TNFR1 is universally expressed on nearly all cell types that recruit several molecules after combining with ligand TNF-α, and then activates downstream signaling, nuclear factor kappa-B (NF-κB) in particular, mostly to activate and exacerbate inflammation. However, TNFR2 is mainly verified to regulate the inflammation reaction level and inhibit cell apoptosis, which could account for why TNFR2 is expressed exclusively on immune cells 90. Bidirectional function of TNF-α is validated to amplify in autoimmune diseases, especially the deleterious effect. During the treatment of autoimmune diseases, particularly exemplified by RA, application of available anti-TNF biologics (monoclonal antibodies, e.g., certolizumab, adalimumab, golimumab and infliximab or soluble TNF receptors, e.g., etanercept) achieves seminal breakthrough since the new start of millennium 91. However, except financial burden and tolerance limitation for few patients, anti-TNF therapy is also associated with multiple, although relatively rare, side effects: increased risk of intracellular infection, particularly reactivation of Mycobacterium tuberculosis, and increased risk of secondary autoimmune manifestations and lymphoma 92.

It is well established that TNF-α plays a critical role in the development of RA and several signaling transduction pathways (NF-κB, JAK/STAT, Akt, p38/MAPK, ERK and Wnt/β-catenin) are identified to be involved in it. To date, in RA, the signaling of NF-κB is pivotal to TNF-α in macrophage, synoviocyte and chondrocyte 93. As an inflammatory biomarker, previous study already indicated the TNF-α-induced expression of PTX3 in synoviocyte of RA patients 61. Currently, the up-regulation of PTX3 by TNF-α in dental pulp cells was also confirmed via the activation of NF-κB signaling 94. Similarly, with the activation of NF-κB signaling in intestinal ischemia and reperfusion injury, PTX3 was demonstrated to be a sensor that promotes the production of inflammatory mediators such as TNF-α and chemokine CXCL1 95. However, the presence of PTX3 was reported to down-regulate IL-1β, TNF-α and MCP-1 expression through repressing the NF-κB signaling activation in macrophages, whilst the detectable concentrations of transforming growth factor-β (TGF-β) was examined 45.

### IL-1β

IL-1β plays a critical role in the degradation of articular cartilage by stimulating both synovial fibroblasts and chondrocytes to secrete MMPs, cathepsins, and mast cell proteinases. Furthermore, in rheumatoid synovium, the upregulation of IL-1β promotes the expression of MMPs, which thereby exacerbates synovial inflammation, increasing joint destruction and bone resorption 96. Interestingly, several studies demonstrated that IL-1β mediated the expression of PTX3 in RA. However, in macrophages, the production of IL-1β, TNF-α and MCP-1 are reduced significantly in the presence of PTX3. Thus, PTX3 may reflect the degree of inflammation indirectly 97.

### IL-6

Among abundant cytokines in circulation during inflammation, IL-6 exerts a powerful systemic effect, which results in common complications in RA patients, including changes in cholesterol metabolism, atherosclerosis and even mood disorders 98. Neiman *et al* had shown that IL-6 may serve as a possible marker for predicting structural damage 99. IL-6, associated with disease progression and joint destruction, is greatly increased in the synovial fluid of RA patients 100. Dessein *et al* indicated that IL-6 is closely related to the biomarkers of endothelial dysfunction due to inflammatory cytokines released from inflamed joints in RA 101. Relevant reports also identified that the concentration of IL-6 can be used as an assessment of the degree of cardiovascular disease in RA 102. However, PTX3 is indirectly affected by the expression of IL-6 103. Therefore, PTX3 and IL-6 are not directly interrelated, but IL-6 may indirectly induce the expression of PTX3 to promote the formation of inflammatory microenvironment of RA 104.

### TGF-β

The immunosuppressive effects of TGF-β have long been emphasized. Meanwhile, RA, an inflammatory disease targetting joints, is caused by abnormal responses of T and B cells. It has been demonstrated that the levels of IL-6, IL-17 and TGF-β in RA patients' peripheral blood serums are significantly elevated 105. The development of TGF-β in RA seems to be determined by the anatomic background of cytokine signaling. Also, the modulation of TGF-β activity could be a potential therapy for Th17-mediated RA 106. Luchetti *et al* reported that TGF-β down-regulated the expression of PTX3 mRNA in RA synoviocytes 61. PTX3 has a potential relationship with TGF-β in RA, which might shed light on the treatment of RA.

### P-selectin

P-selectin is responsible for the interaction of leukocytes and endothelial cells, and promotes tissue damage in antigen-induced arthritis 107. The expression of PTX3 is strongly attributed to the adhesion of monocytes to activated endothelial cells stimulated by TNF-α, which may rely on the interaction between PTX3 and P-selectin 108. Simultaneously, the interaction between P-selectin and PTX3 was identified as the negative feedback loop for recruitment of leukocytes, and P-selectin knockout mice were refractive to PTX3-evoked endothelial dysfunction 109. Meanwhile, Livija Deban *et al* reported the identification of interaction between P-selectin and PTX3, and further elucidated the mechanisms that PTX3 bound to P-selectin via its N-linked glycosidic moiety encoded by the third exon 110. The results may display the role of PTX3 to suppress leukocyte recruitment and inflammation in a P-selectin-dependent way. Herein, it could be speculated that PTX3 may perform an essential role during the onset of RA through acting on endothelial cells and Treg with P-selectin.

## Conclusion and Perspectives

**Figure 2:**
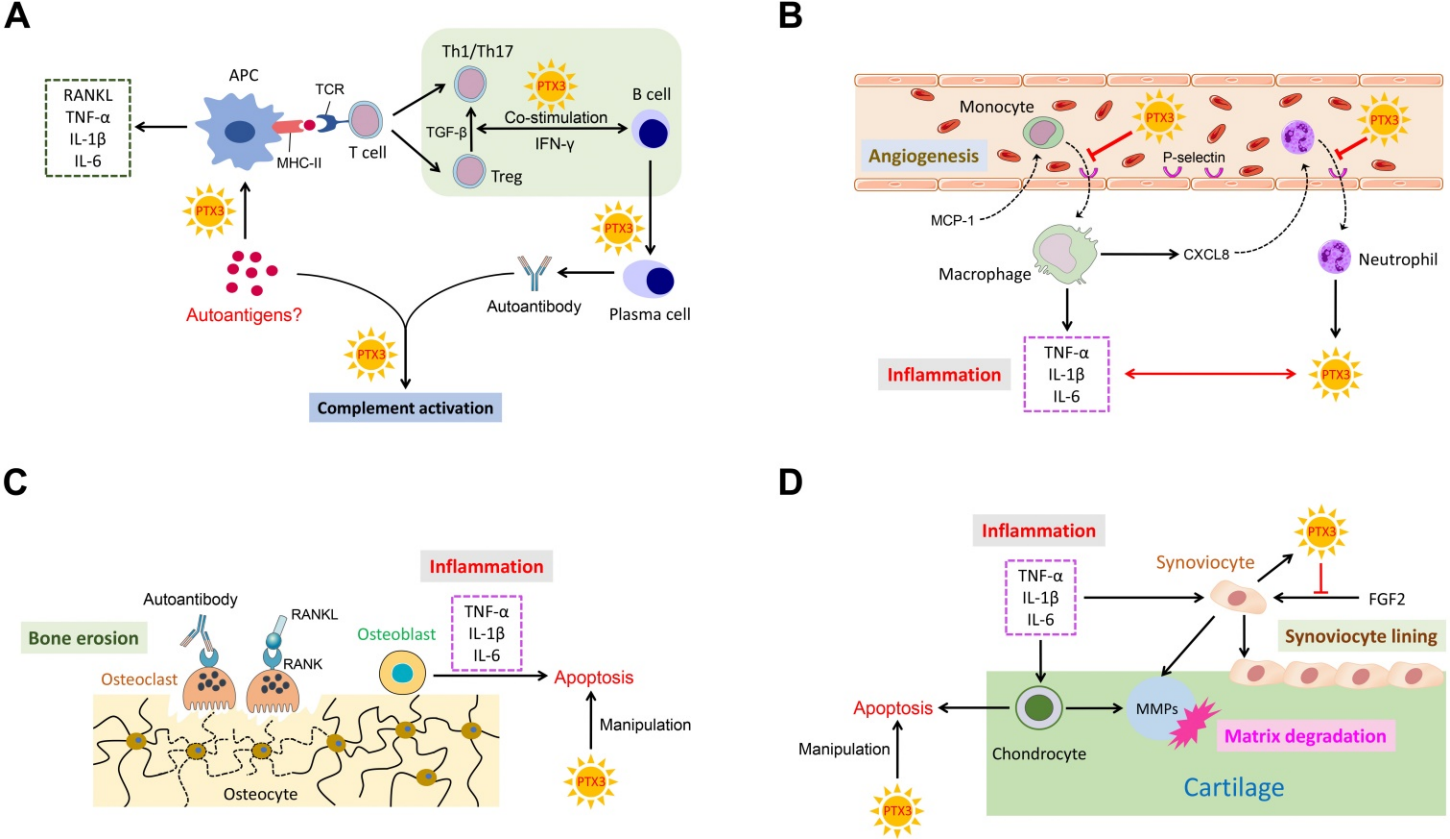
** The ensemble of PTX3 with related cell types and cytokines in RA. (A)** PTX3 promotes autoantigens recognition by APCs, mediates T cells differentiation and activation, regulates the co-stimulation of B cells with T cells, and activates complement system by interacting with the antigen-antibody complex. **(B)** PTX3 inhibits the recruitment of inflammatory cells by targeting on adhesion molecules and interacts with inflammatory cytokines. **(C)** PTX3 manipulates apoptotic osteoblasts. **(D)** PTX3 manipulates apoptotic chondrocytes and antagonizes the function of FGF2 for synoviocyte cell lining.

PTX3 is known to play an indispensable role in several pathological conditions. Conspicuously, as a messenger, the elevated expression of PTX3 is necessary for occurrence and development of inflammation. Importantly, RA is an intricate systemic disorder that typically characterized by the co-function of multiple related cell types in affected synovial joints. Cardinal cell types implicated in RA mainly comprise synoviocyte, chondrocyte, macrophage, dendritic cell, osteoblast, osteoclast, neutrophil and lymphocyte. Relevant cells in RA pathogenesis also function in conjunction with secreted cytokines. Consequently, we depict a schematic representation to summarize these potential interactions with PTX3 involved in RA **(Figure 2)**.

**Figure 3:**
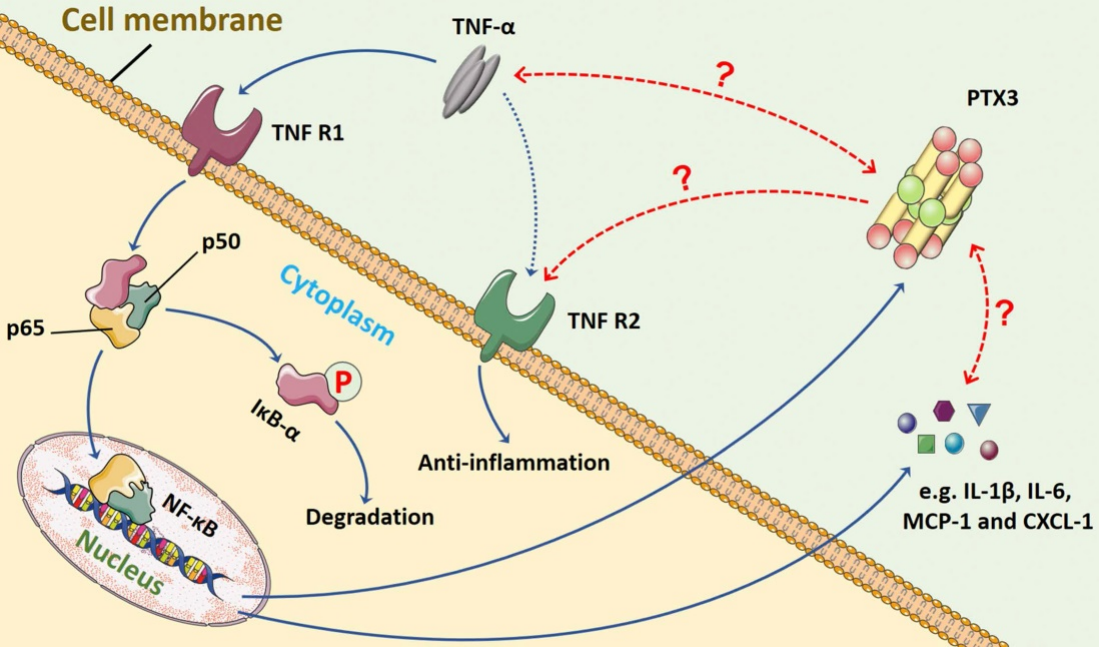
** The possible feedback loop between PTX3 and TNF-α *via* NF-κB signaling pathway in inflammation.** In the progression of inflammation, including RA, the pro-inflammatory cytokine TNF-α mainly binds to TNFR1 that activates NF-κB signaling. The major step is the translocation of the dimer (p50 and p65) into nucleus once IκB-α is inactivated and degraded. The transduction of downstream genes mediated by TNF-α involves abundant inflammatory cytokines (e.g. IL-1β and IL-6) and chemokines (e.g. MCP-1 and CXCL-1). Also, the production of biomarker PTX3 is stimulated by TNF-α. However, whether the interaction between PTX3 and TNF-α exists remains unveiled. The TNF-α/TNFR/NF-κB/PTX3 axis may contribute to a feedback loop co-regulating inflammation.

In terms of the relationship between TNF-α and PTX3, we presume that a feedback loop might exist between them, and the crosstalk may commonly manipulate inflammation progression through NF-κB signaling pathway **(Figure 3)**. The TNF-α/TNFR/NF-κB/PTX3 axis may contribute to a novel therapeutic target for RA.

**Figure 4:**
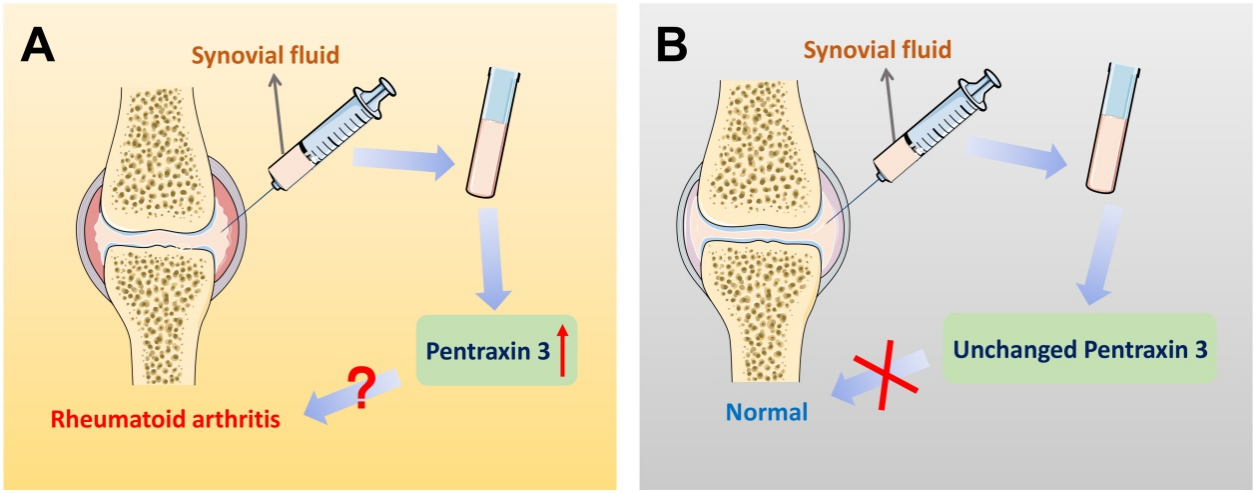
** Pentraxin 3 in synovial fluid might be regarded as a biomarker for the specific differential diagnosis of rheumatoid arthritis.** (A) In the patients of rheumatoid arthritis, there exists a significantly higher level of PTX3 in joint synovial fluid, compared to OA controls. Also, when the expression of PTX3 is abnormally up-regulated in an examinee's synovial fluid, the diagnosis of the disease is likely to be rheumatoid arthritis. (B) In contrast, a lower level of PTX3 is observed in normal people. However, in synovial fluid, when the production of PTX3 remains unchanged, there is no evidence indicating health but at least rheumatoid arthritis may be ruled out.

On account of the current non-specific markers for differential diagnosis of RA, further interpretations of precise markers are required. Therefore, inflammatory molecule PTX3 draws attention. Different expression of PTX3 in synovial fluid between RA patients and the control group is significantly available. Correlations could be identified between different stages of RA progression and the elevated expression of PTX3. The expression of PTX3 might be used as a marker for the evaluation of the activity of RA disease from which the clinical differential diagnosis will benefit. Moreover, compared to the conventional diagnostic marker CRP for RA patients, plausible correlation analysis is also given between PTX3 and RA both in serum and synovial fluid **(Table 2)**. In accord with CRP, locally produced PTX3 in synovial fluid is also endowed with the capacity to imply RA inflammation. Additionally, the concentrations of PTX3 in patients with RA that dynamically lined with the disease, compared to the osteoarthritis (OA) normal. In view of the specific presence of PTX3 in synovial fluid, it may be preferable to choose synovial fluid as a determination of RA diagnosis **(Figure 4)**. Notwithstanding, it may also raise the issue of investigating the cause of churned out PTX3 in synovial fluid of RA patients.

## Figures and Tables

**Table 1:** The role of PTX3 in related typical inflammations

Type of inflammation	Role of PTX3	Reference
Cancerous inflammation	The hypermethylation of PTX3 promoter with subsequent diminished PTX3 expression emerges in human esophageal squamous cell carcinoma, selected mesenchymal and epithelial tumors. *Ptx3^-/-^* mice are susceptible to mesenchymal tumor and dermal cancer, accompanied by increased macrophages infiltration, secreted inflammatory cytokines, neovascularization, increased C3 deposition and C5a levels, and lower FH localization. *Ptx3^-/-^* mice with tumors are mostly characterized by *trp53* mutations. The inhibitory role of PTX3 on several tumor cells is prone to rely on FGF2. PTX3 deficiency also promotes migration and invasion of tumor cells in gastric cancer.	29-34
Retinal inflammation	The expression of PTX3 is increased in cultured RPEC when stimulated with inflammatory cytokines or lipid peroxides *in vitro*. The plasma level of PTX3 and its gene expression in RPEC and choroid are both independent of AMD status. PTX3 regulates the retinal inflammation mainly by binding to FHL-1. In an AMD model, mice with enhanced C3a and IL-1β levels in RPEC and macrophage accumulation in the choroid via complement activation show a deficiency of PTX3.	35-38
Metabolic inflammation	In terms of obesity, PTX3 controls angiogenesis by binding to FGF2. PTX3-deficiency induces polarization of M2-like macrophages to inhibit lipid accumulation and metabolic inflammation in obesity, accompanied by increased CD31 and VEGF in adipose tissue.	30, 39-41
Vascular inflammation	The absence of PTX3 promotes neointimal hyperplasia after vascular injury and macrophage infiltration. PTX3 regulates vascular inflammation by interplaying with adhesion molecules on the vessel wall, associated cytokines and chemokines. Elevated levels of PTX3 in a cardiovascular disease might be associated with the severity of the disease.	42-46
Urinary tract inflammation	Rapid expression of PTX3 to recruit neutrophils to fight against bacterial infection is induced by pathogenic E. coli stimulation via inhibition of TLR4-MyD88 pathway. PTX3-deficiency is highly associated with the increased severity of infection and the increased sensitivity to pathological inflammation.	47-50
Airway inflammation	Th17-dominant CD4^+^ T cells mediate airway inflammatory response in mice with PTX3-deficiency when exposed to OVA. The depletion condition of PTX3 is identified to emerge the phenotype of dysregulated IL-2 and reprograming CD4^+^ T cells. PTX3 is churned out in airway smooth muscle bundles and epithelial cell layers by inflammatory cell infiltration. Higher expression of PTX3 induces ASMC migration and promotes airway remodeling.	51-56

**Table 2:** The comparison between PTX3 and CRP

	PTX3	CRP
Category of Pentraxins	Long	Short
Gene localization in human chromosome	3q25	1q23
Gene structure	3 exons separated by 2 introns	2 exons
Source	Dendritic cell, Monocyte, Macrophage, Synoviocyte, Neutrophil, Adipocyte and Fibroblast	Major hepatocyte
Stimulus	TNF-α and IL-1β	IL-6
Major biological process	Inflammation, Innate immunity, Infection and Tumor	Inflammation
Expression in serum of RA patients	Up-regulation	Up-regulation
Expression in synovial fluid of RA patients	Up-regulation	Up-regulation
Association with the severity of RA	Correlation*	Correlation
Expression in serum after RA treatment	Unchanged	Decreased

*The concentrations of PTX3 in both serum and synovial fluid are related with the severity of Stage II-Ⅳ of RA by Sharma *et al*. A positive correlation of PTX3 in synovial fluid with disease severity is shown by Mustafa Serdar *et al*. There is no correlation of PTX3 in blood with RA according to Lieh-bang Liou *et al* and Gia Deyab *et al*. The definition of correlation is provisional on account of the limited current status.

## Abbreviations

RA: rheumatoid arthritis
PTX3: pentraxin 3
CRP: C-reactive protein
SAP: serum amyloid P
PRM: pattern recognition molecule
FH: factor H
TLR: Toll-like receptor
imDC: immature dendritic cell
FGF2: fibroblast growth factor-2
AMD: age-related macular degeneration
C4BP: C4 binding protein
FHL-1: factor F-like protein 1
Y402H: factor H Y402
RPEC: retinal pigment epithelial cell
VEGF: vascular endothelial growth factor
UTI: urinary tract infection
E. coli: Escherichia coli
OVA: ovalbumin
ASMC: airway smooth muscle cell
ACPA: against cyclocitrulline peptide
RF: rheumatoid factor
IL-1β: interleukin-1beta
TNF-α: tumor necrosis factor-alpha
PGE2: prostaglandin E2
H+: protons
SAA: serum amyloid A
LPS: lipopolysaccharide
LDL: low-density lipoprotein
DC: dendritic cell
RANKL: receptor activator of nuclear factor kappa-B ligand
ALP: alkaline phosphatase
MCP-1: monocyte chemoattractant protein-1
ROS: reactive oxygen species
OPG: osteoprotegerin
NF-κB: nuclear factor kappa-B
OA: osteoarthritis
